# Physical inactivity and sedentary behaviours: screening and intervention in primary care, *a prospective*,* multicentre*,* cluster-randomised*,* controlled*,* stepped-wedge study*

**DOI:** 10.1186/s12889-025-25410-4

**Published:** 2025-11-14

**Authors:** Nicolas Pinsault, Sophie Rey, Léo Druart, Agnès Helme Guizon, Romain Debru, Matthieu Roustit, Christophe Pison

**Affiliations:** 1 Département de kinésithérapie, University of Grenoble Alpes, Saint Martin d’Hères, France; 2 TIMC lab, THEMAS team, UMR CNRSUGA 5525, Grenoble, France; 3 Department of Diagnostic Imaging, Warren Alpert Medical School, Brown University, Providence, Rohde Island USA; 4 Medical Expectations Lab, Brown University, Providence, Rohde Island USA; 5 Grenoble IAE-INP, University of Grenoble Alpes, Grenoble, 38000 France; 6 Grenoble INP, CERAG, University of Grenoble Alpes, Grenoble, France; 7 University of Savoie Mont Blanc, IAE Savoie Mont Blanc, Annecy, France; 8 Laboratoire IREGE, University of Savoie Mont Blanc, Annecy, France; 9 Univ. Grenoble Alpes, CHU Grenoble Alpes, Inserm, Grenoble, CIC 1406 France; 10 Inserm, HP2, U1300, University of Grenoble Alpes, Grenoble, France; 11 Laboratoire de Bioénergétique Fondamentale et Appliquée, LBFA, Inserm1055, University of Grenoble Alpes, Saint Martin d’Hères, France; 12 Centre de Pneumologie Henri Bazire, Saint Julien de Ratz, France

**Keywords:** Primary care, General practitioner, Physiotherapist, Physical activity, Physical inactivity, Sedentary lifestyle, Serious game, RCT

## Abstract

**Background:**

Physical inactivity and sedentary behaviours (PiA/SED) are among the major modifiable risk factors for chronic diseases. Behaviour change models for PA can shape personalised interventions leading to sustainable lifestyle changes. We hypothesise that screening for PiA/SED by a general practitioner, followed by a personalised intervention by a physiotherapist, could reduce PiA/SED in inactive adults.

**Methods:**

We designed a prospective, multicentre, cluster-randomised, controlled, step-wedge study. Adult patients without chronic illnesses will be recruited in 8 multi-professional health centres. They will receive educational content on PiA/SED. During the intervention periods, patients will see a physiotherapist for a functional assessment, and an intervention aimed at improving PiA/SED using a serious game. Two follow-up appointments at months 2 and 4 (M2-4) are planned to maintain patient motivation. At M6, a 7-day actimetry will be performed, and at M6-12, questionnaires and semi-structured interviews will close the study. Two primary endpoints will be analysed using a pre-specified hierarchical sequential analysis: the proportion of patients changing PiA/SED at M6. Secondary objectives include: 1-describing changes in PiA/SED at M6 and M12, 2-exploring the link between patient characteristics and changes in PiA/SED, 3-describing participants’ quality of motivation, satisfaction of basic psychological needs, feelings of self-efficacy, perceived levels of vitality and energy, and self-esteem, 4-describing the strategies, barriers and facilitators of behavioural changes, 5-studying the correlation between questionnaires measuring physical activity and actimetry, 6-identifying the perceived barriers and facilitators to implement this care pathway. Assuming that 10% of patients in the control period will improve their PA and that the intervention will increase it by 20%, 160 patients provides 82% power to observe a significant difference.

**Discussion:**

This design will harmonise the skills of all professionals in the field of motivational support for PiA/SED and providing information about the risks associated with PiA/SED. Patients in the intervention group will also receive individual support for behaviour changes related to PiA/SED. Considering public health, this study will contribute to increase primary prevention by healthcare professionals. Finally, this study will assess the effectiveness, adherence, satisfaction of the stakeholders involved in this pathway allowing to consider its implementation in routine primary care.

**SPIRIT 2025 checklist of items:**

see supplement files.

**Trial registration:**

ClinicalTrials n° NCT06678906, https://clinconnect.io/trials/NCT06678906#about-company-tab, first registration October 14, 2024, Trial updated February 05, 2025.

**Supplementary Information:**

The online version contains supplementary material available at 10.1186/s12889-025-25410-4.

## Background

Physical inactivity (PiA) and sedentary behaviour (SED) are major risk factors for several chronic diseases and premature mortality [1–3]. Globally, 7.2% of deaths from all causes and 7.6% of cardiovascular diseases are attributable to PiA based on data collected from 168 countries in 2018 [1,4]. While often associated together, PiA and SED are distinct behaviours, each with their own health effects [5]. This distinction highlights four possible profiles: active yet sedentary, active and non-sedentary, inactive and sedentary, and inactive yet non-sedentary. In France in 2015, a national survey reported low physical activity (PA) and high SED in 37% and 80% of people aged 18 to 79 respectively [6, 7].

As these are modifiable risk factors [8], healthcare professionals, more particularly primary care general practitioners (GP) and physiotherapists (PT) are well positioned to promote physical activity, as shown in a 2022 meta-analysis [9]. However, of 46 randomized controlled trials included, only 14 measured physical activity through devices among which no significant increases in moderate to vigorous physical activity were observed [9]. Additionally, interventions should incorporate well-described and coherent behavioural change models such as the Health Action Process Approach [10], the Multi-Process Action Control in Physical Activity Model [11] and the theory of effort minimisation [12].

We propose that an innovative healthcare pathway, involving opportunistic screening by GPs and short, individualized and pragmatic interventions by PTs based on behavioural change models, can effectively facilitate behaviour changes towards increased PA and reduced SED at six months in inactive adults not already suffering from chronic illnesses.

## Method

### Primary aim and outcomes

The primary objective is to evaluate the 6-month effectiveness of an innovative care pathway that includes opportunistic screening for PiA and SED by a GP, followed by a personalized intervention by a PT on PiA/SED for adult patients not already suffering from chronic illnesses reimbursed by the French Social Security system.

Two main criteria will be analysed, according to a predefined hierarchical sequential analysis as follows: (1) the proportion of patients changing PA category (inactive, moderately active, very active), measured by actimetry, between inclusion (M0) and the sixth month (M6), to a higher-ranking category; (2) the proportion of patients changing SED category (sedentary, non-sedentary), measured by actimetry, between M0 and M6, to a lower-ranking category.

PA and SED will be measured at inclusion, M0 and at M6 post-intervention from a 7-day actimetry performed with the wGT3X-BT device, Actigraph, Pensacola, USA. PA profiles are defined as: inactive < 600 MET.min/week; moderately active < 1200 MET.min/week; very active ≥ 1200 MET.min/week^8^. Sedentary behaviour is defined as time spent lying or sitting without sleeping with energy expenditure close to resting (≤ 1.5 METS) [8]. The threshold of 9 h/day of sedentary behaviour is considered due to its association with an increased all-cause mortality risk [13].

### Secondary aims and outcomes

Secondary objectives include: (1) describing the evolution in PA and SED of patients at M6 and M12 for behaviour maintenance; (2) exploring the relationship between patient characteristics, such as age, sex, socioeconomic status, environment, living area, and occupation, and the change at M6 and maintenance at M12 of PiA and SED; (3) describing the quality of motivation, satisfaction of basic psychological needs, sense of personal effectiveness, perceived vitality and energy level, and self-esteem; (4) describing at M6 and M12 the strategies, barriers, and levers to behavioural change through semi-structured interviews, coupled with a qualitative survey *via* semi-structured interviews on a volunteer subsample of 25% of participants, who may or may not have benefited from the intervention at M6 and M12; (5) studying the relationship between the ONAPS-PAQ questionnaire [14] results and the 7-day actimetry among the inactive and/or sedentary population category; (6) identifying perceived barriers and facilitators to implementation in primary care at 12 months among different stakeholders, including GPs, PTs, and patients.

Outcomes for PA and SED will be measured at M6 by a 7-day actimetry and at M12 by the ONAPS-PAQ questionnaire [14]. Secondary aim number 3 will be measured through five questionnaires: *Echelle de Motivation envers l’Activité Physique en contexte de Santé* (EMAPS) [15], scale measuring basic psychological needs [16], scale measuring perceived competence to engage in a personalized physical activity program [17], scale measuring perceived vitality and energy level [18], and the self-esteem scale [19] at M0, M6, and M12.

### Population

The study involves adults not suffering from chronic illnesses reimbursed by the French Social Security system, receiving outpatient primary care from one of the 8 participating multi-professional health centres (MPHC). These individuals are classified as inactive, regardless of being sedentary or non-sedentary, based on the ONAPS-PAQ questionnaire and ultimately by actimetry. Participants signed an informed consent to be part of the study and were confirmed inactive after the analysis of 7-day accelerometry data.

### Setting and design

We designed a prospective, multicentre, randomised, cluster-controlled step-wedge [20] study in 8 MPHC dedicated to primary care within Auvergne Rhône-Alpes area in France.

Pre-Inclusion: during a routine appointment with a GP at a participating MPHC, the patient is invited to complete the ONAPS-PAQ questionnaire using a REDCap survey via a QR code on a study information poster in the waiting room or by the doctor’s secretary. During the consultation, after verifying the inclusion criteria thanks to the questionnaire results, the GP presents the study to patients classified as “inactive” according to the ONAPS-PAQ. At this stage, the patient is further tested for eligibility by completing a 7-day actimetry for definitive inclusion. Consent to participate of each participant is collected accordingly to the National Biomedical Ethics Committee North-West 3 clearance, under the number N° ID/RCB : 2024-A00983-44.

Inclusion: at home, the patient completes the questionnaires, scales online, and provides demographic information (self-entry using the REDCap software). The patient receives the accelerometer at home along with all necessary instructions. They are required to wear it for 8 consecutive days (accelerometry is measured over 7 days) and simultaneously maintain a log detailing the start and end times of wear, any periods of non-wear, and any naps taken during the day.

**Fig. 1:**
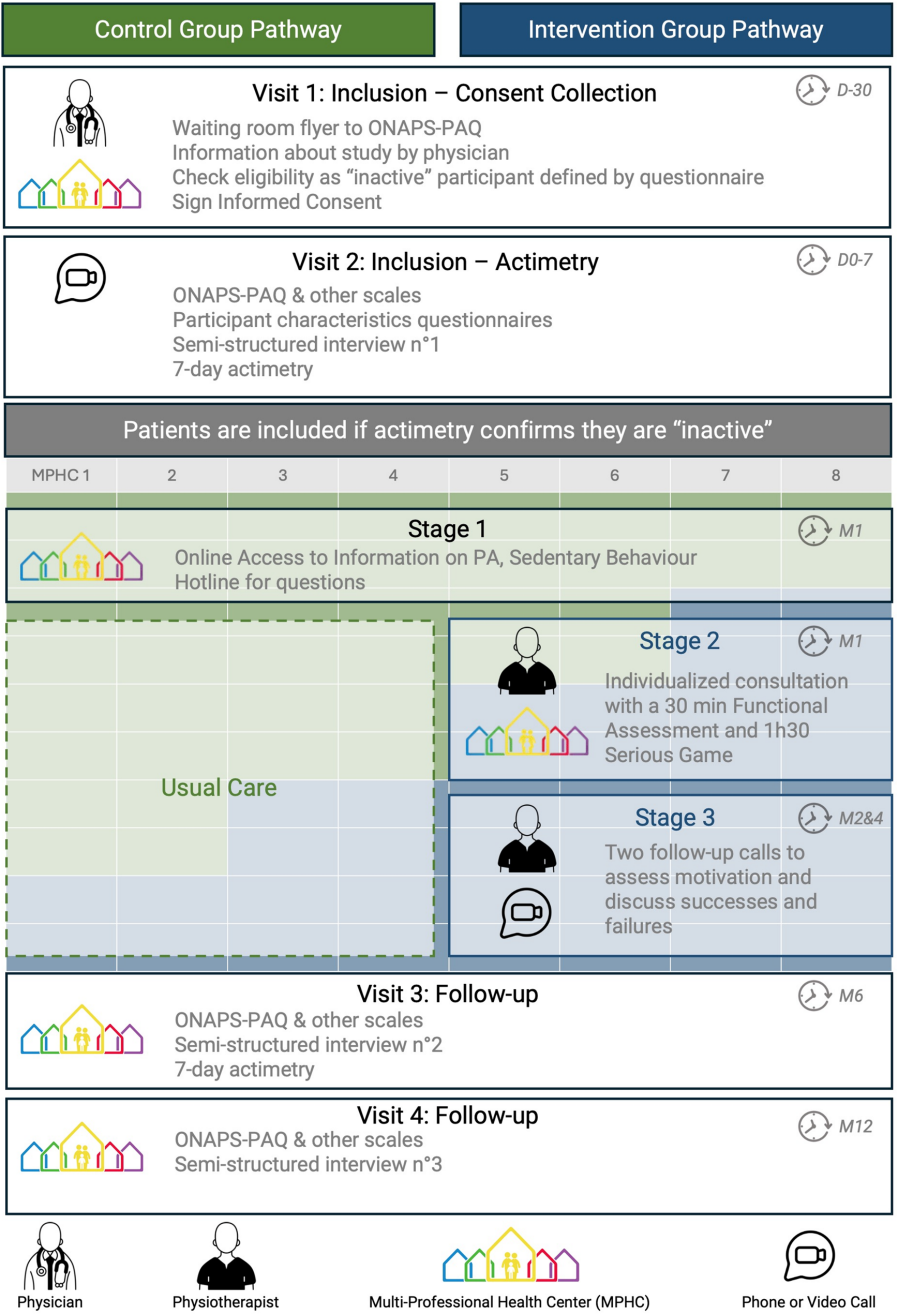
Work flow of the study and and the cluster-randomised stepped-wedge design. The Stepped-Wedge design means that at the start all 8 MPHC will deliver the control pathway for the first 2 months as represented with the table in green and blue below. Then, every 2 months 2 new MPHC will switch to the interventionnal pathway. Finally, for the last 2 months, all MPHCs will deliver the interventionnal pathway

While all enrolled patients will receive standard information on PA and SED (intervention step 1), only patients in the intervention group will receive a functional assessment and personalized behaviours change intervention. The intervention consists of a serious game (step 2) during an individual appointment with a PT at the MPHC and remote coaching at M2 and M4 (step 3), as shown in Fig. 1. During follow-up visits at M6 and M12, patients will be required to complete questionnaires and scales, and some will also participate in semi-structured interviews. The objectives and questions for the semi-structured interviews will be the same at M6 and M12, with the same participant being interviewed at both M6 and M12 by the same investigator. The visit conducted at M12 will be the end-of-study visit.

### Interventions and comparisons, Fig. 1 and Table 1

**Table 1.**
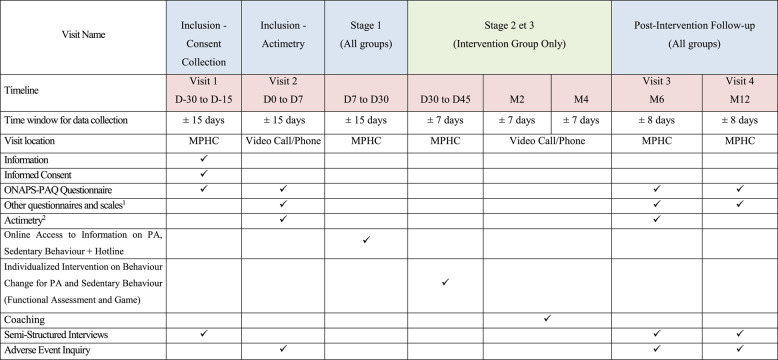
*Summary of outcomes and data collection. In blue visits that concerne all groups, in green visits that are only for the intervention group*

^1^ Echelle de Motivation envers l'Activité Physique en contexte de Santé (EMAPS), scale measuring basic psychological needs, scale measuring perceived competence to engage in a personalized physical activity program, scale measuring perceived vitality and energy level, and the self-esteem scale

In Stage 1, all participants are informed about benefits of PA and risks of SED [21] through video capsules made available to all included patients throughout the study. The videos cover definitions of PiA and SED, the short-, medium-, and long-term health impacts of regular PA, the health impact of SED and how to move more and move for longer. A hotline will address any patient inquiries.

The objectives of Stage 2, reserved for the intervention group, are to assess the functional abilities of patients to personalize the support provided. This involves a 2-hour individual appointment with a trained PT from the MPHC and the patient. The Stage 2 appointment includes a 30-minute physiotherapy functional assessment (details of the assessment provided in supplementary material-B) and a 90-minute serious game co-constructing actions through 4 phases of gameplay (details of the game provided in supplementary material). The first phase of the game, the diagnostic phase, focuses on a motivational diagnosis of behaviour change regarding PA and SED and lasts 30 min. Then, the projection phase involves sensitization to action planning and anticipation of obstacles and lasts 30 min. Finally, the commitment phase focuses on the implementation and planning of challenges related to PA and SED for the remaining 15 min.

Stage 3 consists of two follow-up sessions at M2 and M4. The aim of these two 30-minute teleconsultation sessions are to highlight the patient’s successes and failures over the past period and work on the causal attribution (internal/external - modifiable/non-modifiable - controllable/uncontrollable) of successes and failures with the patient. The objectives defined in stage 2 are adapted if necessary and discussed. The PT providing Stage 2 will be the same to provide the M2 and M4 teleconsultation follow-ups for this patient (Stage 3).

#### Statistical analysis

A stepped-wedge design (Fig. 1) with 5 time periods and 4 steps, involving 8 centres transitioning from the control period to the experimental period (2 centres per step), with an average of 20 patients per centre (approximately 4 patients per centre per time period), results in a total sample size of 160 patients.

This design provides 82% power to detect a significant difference between the groups at the first level of the hierarchy (PASS v22, NCSS, LLC, Kaysville, Utah, USA). This calculation is based on the method described by Hemming et al. [22], which uses a linear mixed-effects model with time as a fixed factor at T levels and inter-cluster variation modelled as a random effect. The test statistic employed is a two-sided z-test. Our assumptions leading to this conclusion are that the intervention increases the proportion of patients changing their physical activity profile, corresponding to the first level of the hierarchy, by 20% (in absolute value), assuming that 10% of patients in the control period will experience an improvement. The intraclass correlation coefficient (ICC) is 0.01, and the significance level of the test is 0.05.

For each of the two groups, qualitative variables will be described by their count and percentage, while quantitative variables will be characterized by their mean and standard deviation, or median and interquartile range if they do not follow a normal distribution. The normality of quantitative variables will be assessed by graphical inspection and the Shapiro-Wilk test. The primary analysis does not plan to impute missing data. However, we propose the following strategy currently recommended [23] to minimize the impact of missing data as much as possible: reduce attrition to a minimum by limiting constraints (number of site visits, etc.), continue patient follow-up and outcome collection even in cases of protocol deviations, and consider sensitivity analyses on the per-protocol population, as well as on the ITT population after replacing missing data by multiple imputation methods, provided the assumption of missing at random (MAR) is credible.

A detailed statistical analysis plan will be drafted before the database lock. It will take into account any protocol amendments or unexpected events that occurred during the study and impacted the analyses presented above. The planned analyses may be supplemented in alignment with the study objectives.

## Discussion

Healthcare professionals in primary ambulatory care, including GPs and PTs, are already involved in tertiary prevention and can also contribute to primary and secondary prevention, particularly in the domain of PA^9^. With their expertise in assessment, support, and goal customization, they can increase opportunities for prevention before patients develop long-term conditions.

The preventive dimension of the study, the personalized and participatory nature of the intervention, and the prediction of success/failure through data analysis allow to fulfil the requirements of 4P medicine—preventive, personalized, predictive, and participatory—in primary ambulatory care.

This project is original, innovative, and promising due to its pragmatism and anticipated feasibility in primary ambulatory care, the involvement of healthcare providers in primary-secondary prevention, the multi-professional collaboration involving screening/motivation by the GP and intervention by the PT, an intervention based on current theoretical behaviour change models and a pathway combining face-to-face and online support allowing personalisation of the intervention by a trained PT. Additionally, the use of a stepped-wedge design allows to harmonise the competence of all professionals in motivational support for PA and provide information about the risks associated with PiA/SED. This has two main benefits. First, it allows for a strict control of the effect of the personalised intervention given all participants will have access to information about the benefits of PA and risks of PiA/SED. Second, at the end of the trial, all investigators will have benefited from specialised training in primary prevention of SED/PiA. This would not have been the case in a parallel cluster randomised trial.

Patients will truly be active participants in their behaviour change as the intervention aims to meet the three fundamental psychological needs: need for autonomy: the patient sets their own challenges and activities; need for social relatedness: support from trained and supportive professionals; and need for competence: adapting challenges to the patient’s skills and defining intermediate challenges will help maintain the patient in a context of success.

## Supplementary Information

Supplementary Material 1.

## Data Availability

No datasets were generated or analysed at this stage.
