# Supplementary material for: Physical inactivity and sedentary behaviours: screening and intervention in primary care, a prospective, multicentre, cluster-randomised, controlled, stepped-wedge study
Source: BMC Public Health. 2025 Nov 14;25:3951. doi: 10.1186/s12889-025-25410-4 (PMC12619489; doi:10.1186/s12889-025-25410-4)
Supplement: Supplementary file 1 — Supplementary Material 1. [file 12889_2025_25410_MOESM1_ESM.docx]

***Supplementary materials***

A - SPIRIT 2025 checklist of items to address in the randomized trial protocol “Physical inactivity and sedentary behaviours: screening and intervention in primary care”

B - Physiotherapy Functionnal Assessment

C - Serious game description

1. **SPIRIT 2025 checklist of items to address in the randomized trial protocol** **“Physical inactivity and sedentary behaviours: screening and intervention in primary care”**

| **Section / Topic** | **No** | **SPIRIT 2025 checklist item description** | **Reported on page no.** |
| --- | --- | --- | --- |
| **Administrative information** | | |  |
| Title and structured summary | 1a | Title stating the trial design, population, and interventions, with identification as a protocol | 1 |
|  | 1b | Structured summary of trial design and methods, including items from the World Health Organization Trial Registration Data Set | 2 |
| Protocol version | 2 | Version date and identifier : 2024-A00983-44_Protocole_final_V2.0_20241205_IPSEDI |  |
| Roles and responsibilities | 3a | Names, affiliations, and roles of protocol contributors | 1, 11 |
|  | 3b | Name and contact information for the trial sponsor | 11 |
|  | 3c | Role of trial sponsor and funders in design, conduct, analysis, and reporting of trial; including any authority over these activities | 11 |
|  | 3d | Composition, roles, and responsibilities of the coordinating site, steering committee, endpoint adjudication committee, data management team, and other individuals or groups overseeing the trial, if applicable | 11 |
| **Open science** | | |  |
| Trial registration | 4 | Name of trial registry, identifying number (with URL), and date of registration. If not yet registered, name of intended registry | 3 |
| Protocol and statistical analysis plan | 5 | Where the trial protocol and statistical analysis plan can be accessed | 3 |
| Data sharing | 6 | Where and how the individual de-identified participant data (including data dictionary), statistical code, and any other materials will be accessible | 3 |
| Funding and conflicts of interest | 7a | Sources of funding and other support (e.g., supply of drugs) | 11 |
|  | 7b | Financial and other conflicts of interest for principal investigators and steering committee members | 1 |
| Dissemination policy | 8 | Plans to communicate trial results to participants, healthcare professionals, the public, and other relevant groups (e.g., reporting in trial registry, plain language summary, publication) | 11 |
| **Introduction** | | |  |
| Background and rationale | 9a | Scientific background and rationale, including summary of relevant studies (published and unpublished) examining benefits and harms for each intervention | 4 |
|  | 9b | Explanation for choice of comparator | 4 |
| Objectives | 10 | Specific objectives related to benefits and harms | 4-6 |
| **Methods: Patient and public involvement, trial design** | | |  |
| Patient and public involvement | 11 | Details of, or plans for, patient or public involvement in the design, conduct, and reporting of the trial | 6-9 |
| Trial design | 12 | Description of trial design including type of trial (e.g., parallel group, crossover), allocation ratio, and framework (e.g., superiority, equivalence, non-inferiority, exploratory) | 8-9 |
| **Methods: Participants, interventions, and outcomes** | | |  |
| Trial setting | 13 | Settings (e.g., community, hospital) and locations (e.g., countries, sites) where the trial will be conducted | 6 |
| Eligibility criteria | 14a | Eligibility criteria for participants | 6-7 |
|  | 14b | If applicable, eligibility criteria for sites and for individuals who will deliver the interventions (e.g., surgeons, physiotherapists) | 6-7 |
| Intervention and comparator | 15a | Intervention and comparator with sufficient details to allow replication including how, when, and by whom they will be administered. If relevant, where additional materials describing the intervention and comparator (e.g., intervention manual) can be accessed | 4-9 |
|  | 15b | Criteria for discontinuing or modifying allocated intervention/comparator for a trial participant (e.g., drug dose change in response to harms, participant request, or improving/worsening disease) | NA |
|  | 15c | Strategies to improve adherence to intervention/comparator protocols, if applicable, and any procedures for monitoring adherence (e.g., drug tablet return, sessions attended) | 6-9 |
|  | 15d | Concomitant care that is permitted or prohibited during the trial | NA |
| Outcomes | 16 | Primary and secondary outcomes, including the specific measurement variable (e.g., systolic blood pressure), analysis metric (e.g., change from baseline, final value, time to event), method of aggregation (e.g., median, proportion), and time point for each outcome | 4-6 |
| Harms | 17 | How harms are defined and will be assessed (e.g., systematically, non-systematically) | NA |
| Participant timeline | 18 | Time schedule of enrollment, interventions (including any run-ins and washouts), assessments, and visits for participants. A schematic diagram is highly recommended (see Figure) | Table 1 and Fig. 1 |
| Sample size | 19 | How sample size was determined, including all assumptions supporting the sample size calculation | 8-9 |
| Recruitment | 20 | Strategies for achieving adequate participant enrollment to reach target sample size | 8 |
| **Methods: Assignment of interventions** | | |  |
| Randomization: |  |  |  |
| Sequence generation | 21a | Who will generate the random allocation sequence and the method used | 6-7 |
|  | 21b | Type of randomization (simple or restricted) and details of any factors for stratification. To reduce predictability of a random sequence, other details of any planned restriction (e.g., blocking) should be provided in a separate document that is unavailable to those who enroll participants or assign interventions | 6-7 |
| Allocation concealment  mechanism | 22 | Mechanism used to implement the random allocation sequence (e.g., central computer/telephone; sequentially numbered, opaque, sealed containers), describing any steps to conceal the sequence until interventions are assigned | 6-7 |
| Implementation | 23 | Whether the personnel who will enroll and those who will assign participants to the interventions will have access to the random allocation sequence | 7 |
| Blinding | 24a | Who will be blinded after assignment to interventions (e.g., participants, care providers, outcome assessors, data analysts) | 8-9 |
|  | 24b | If blinded, how blinding will be achieved and description of the similarity of interventions | NA |
|  | 24c | If blinded, circumstances under which unblinding is permissible, and procedure for revealing a participant’s allocated intervention during the trial | 7 |
| **Methods: Data collection, management, and analysis** | | |  |
| Data collection methods | 25a | Plans for assessment and collection of trial data, including any related processes to promote data quality (e.g., duplicate measurements, training of assessors) and a description of trial instruments (e.g., questionnaires, laboratory tests) along with their reliability and validity, if known. Reference to where data collection forms can be accessed, if not in the protocol | 8 |
|  | 25b | Plans to promote participant retention and complete follow-up, including list of any outcome data to be collected for participants who discontinue or deviate from intervention protocols | 6-9 |
| Data management | 26 | Plans for data entry, coding, security, and storage, including any related processes to promote data quality (e.g., double data entry; range checks for data values). Reference to where details of data management procedures can be accessed, if not in the protocol | 6-9 |
| Statistical methods | 27a | Statistical methods used to compare groups for primary and secondary outcomes, including harms | 8-9 |
|  | 27b | Definition of who will be included in each analysis (e.g., all randomized participants), and in which group | 7 |
|  | 27c | How missing data will be handled in the analysis | 9 |
|  | 27d | Methods for any additional analyses (e.g., subgroup and sensitivity analyses) | 9 |
| **Methods: Monitoring** | | |  |
| Data monitoring committee | 28a | Composition of data monitoring committee (DMC); summary of its role and reporting structure; statement of whether it is independent from the sponsor and funder; conflicts of interest and reference to where further details about its charter can be found, if not in the protocol. Alternatively, an explanation of why a DMC is not needed | see proto-col |
|  | 28b | Explanation of any interim analyses and stopping guidelines, including who will have access to these interim results and make the final decision to terminate the trial | 9 |
| Trial monitoring | 29 | Frequency and procedures for monitoring trial conduct. If there is no monitoring, give explanation | see proto-col |
| **Ethics** | | |  |
| Research ethics approval | 30 | Plans for seeking research ethics committee/institutional review board approval | 12 |
| Protocol amendments | 31 | Plans for communicating important protocol modifications to relevant parties | 9 |
| Consent or assent | 32a | Who will obtain informed consent or assent from potential trial participants or authorized proxies, and how | 6-7 |
|  | 32b | Additional consent provisions for collection and use of participant data and biological specimens in ancillary studies, if applicable | NA |
| Confidentiality | 33 | How personal information about potential and enrolled participants will be collected, shared, and maintained in order to protect confidentiality before, during, and after the trial | Table 1 |
| Ancillary and post-trial care | 34 | Provisions, if any, for ancillary and post-trial care, and for compensation to those who suffer harm from trial participation | NA |

**B – Physiotherapy functional assessment - 30 minutes**

- Question the patient's medical and surgical history

- Assessment of fatigue

- Pain assessment

- Assessment of the link made by the patient between pain - PA - SED

- Assessment of passive and active mobility and motor control

- Qualitative appraisal during the passage on the floor / survey of the floor from the previous exercise

- Assessment of balance: One Leg balance test: YO and YF unipodal balance test

- Assessment of physical condition: 1 minute chair lift test

- Assessment of perceived exertion using the Borg Scale at the end of the 1-minute chair-lift test.

Rationale for functional assessment. The aim of the functional assessment is to modulate the way in which the serious game is approached, particularly in terms of objectives, depending on the functional abilities, activity limitations and participation restrictions identified during the physiotherapy diagnostic assessment. The efficiency of behaviour change should be improved by defining SMART (Specific, Measurable, Attainable, Realistic and Timed) objectives.

At the end of the functional assessment, the individual session with the physiotherapist continues in the form of the serious game described above.

**C – Serious game based on the co-construction of actions - 1h30**

**‘OBJECTIVE… ACTIVE!’**

**Rules of the game and instructions**

*
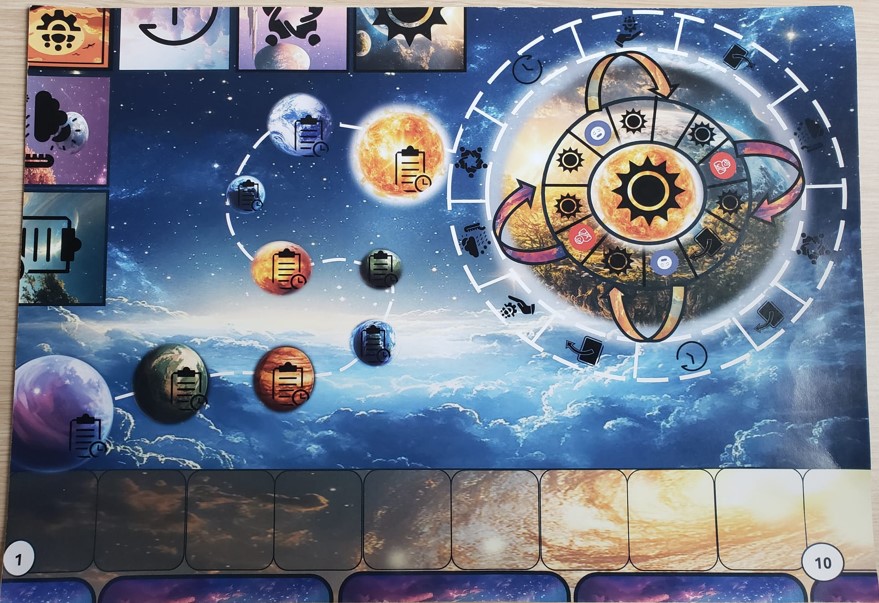
*

***Game board***

**General instructions**

**Time available and set-up:** you have approximately 1 hour to play with the subject. During certain phases of the game, the physiotherapist (PT) may decide to speed up or slow down the game. The PT and the subject face each other; at the start of the game, the subject sits on the side of the visual scale.

**Involvement of the subject**: the subject should participate as much as possible in the game. Let them handle the cards and counters, and read the cards out loud if possible (with the exception of the cards in the ‘I'm going’ stage, which are read by the PT

**Involvement of the physiotherapist**: Apart from the ‘FLIGHT PLAN’ planning phase, when the physiotherapist plays with the subject to set the pace of the game, the physiotherapist intervenes as little as possible. No judgement on the answers given, no suggestions... the physiotherapist may suggest that the subject clarify certain points and tries to answer any questions the subject may have. For the multiple-choice questions on the back of the cards in the ‘I'm going’ phase, the physiotherapist may, if necessary, refer to the theoretical elements in the capsules to help the subject answer.

***Explanation to be given to the subject at the start of the game, in bold***

**We're going to play this OBJECTIVE ACTIVE game together to talk about physical activity and a sedentary lifestyle. Throughout the game, you will have to reach the SUN, which symbolises long-term physical activity for good health. To do this, you need to complete the various stages. The game has 4 stages (START / FLIGHT PLAN / APPROACH / SET UP).**

**During the first stage, ‘I AM GOING’, you are the one who is concerned, and this concerns this part of the game board (show the zone). During this stage, the questions may lead you to reflect a little on yourself to find out why you are embarking on this ‘ACTIVE OBJECTIVE’.**

**Next, we're going to play with a fictitious inactive character who needs to be made to move more! You can invent a life for him or even superpowers to help him get through the different stages. In the second phase of the game, you'll have to define the ‘FLIGHT PLAN’ and plan your character's activities. The ‘APPROACH’ is the third stage, where you'll have to help your character avoid the little traps that could prevent him from carrying out the planned activity. In the final phase of the game, the ‘SETTING THE SCENE’ around the SUN, your character will have to plan for the longer term to ensure that the changes towards more physical activity and a less sedentary lifestyle are sustainable.**

**Are you ready? Do you have any questions?**

**PREAMBLE - Dream card [5 minutes]**

**‘What would you like to be able to do in 5 years' time in terms of physical activity? We're talking about a dream here, not necessarily an objective that can be achieved in reality!**

The subject explains their dream to the physio and symbolises it graphically in the box on the front of the folded dream card. The dream may or may not be realistic.

The dream card is placed on the table next to the game board

**GAME PHASE 1: ‘I'M GOING’ - motivational assessment**

**‘Now I'm going to read out the statements on these cards. As I put them on the table, you should take the cards that speak to you the most and that correspond to your current state of mind. In the end, you'll need to keep 5 cards, but if you take more, that's OK, you can sort them out later.**

The physiotherapist reads the front of the 19 cards slowly and clearly to the subject and places them face down. As the cards are read, the subject picks the cards that correspond to him or her. At the end of this phase, the subject must keep the 5 cards that correspond most to him or her. These are placed on the deck by the subject in the spaces reserved (face up = recto).


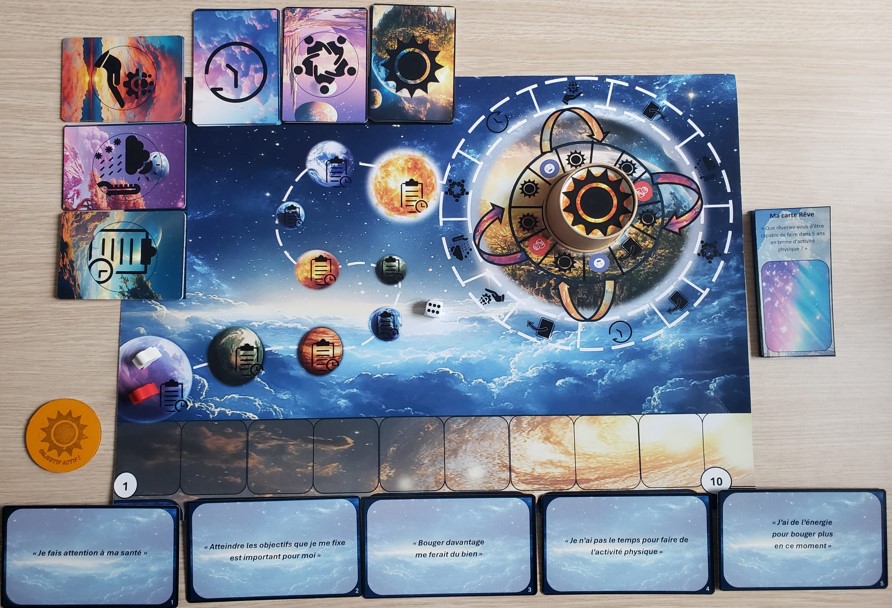


Game board with Dream card on the right and I am going cards at the bottom

Throughout the game, when players find the ‘turn over a card’ logo, the subject chooses one of the cards from the ‘I AM GOING’ stage, reads it and turns it over to play it. Once played, the card is placed back on the back of the board. If there is an ‘I GO’ card that has not been turned over at the end, the physio invites the subject to turn it over and play it at the end of the game.

If necessary, sheets of paper and a pen are available in the game box to play certain cards. If the answers to the multiple-choice questions are wrong, they can be corrected immediately by the physiotherapist and discussed again if necessary (the physiotherapist can use the video clips that have been watched).

**PHASE 2 OF THE GAME: "THE FLIGHT PLAN" – Planning**

**"We will now move on to the phase where we play with a fictional character. This character is inactive and sedentary, and your mission is to guide them toward more physical activity and less sedentary behavior. You can choose a token and create a life for them! Decide on their name, age, job, family situation, and even their place of residence if you like! I'll do the same with my character. In this phase of the game, we're taking them on a journey to the SUN."**

Both players (physiotherapist and participant) choose a token and create a life for their character, including age, socio-professional situation, family situation, and place of residence. Some participants might want to play with a character that closely resembles them! Let them do so, but continue to refer to "their character." The physiotherapist should try to choose a character quite different from the one selected by the participant.

Each space must be crossed one by one by each player (participant and physiotherapist). Planning cards are drawn and played by both players alternately. Depending on how easily the participant interacts, the physiotherapist may start first to facilitate interaction.

When a "flip a card" card is drawn, it is up to the participant to play it, and the tokens do not advance.

**PHASE 3 OF THE GAME: "THE APPROACH" – Anticipating Obstacles**

**"Great, your character has reached the third stage, THE APPROACH! You'll need to figure out how to help your character overcome everyday obstacles that might prevent them from implementing what's outlined in their 'FLIGHT PLAN.' Now, you'll play alone with the dice, and I'll move this token on the visual scale based on the effectiveness of your responses to help your character move forward! You'll quickly understand ;-) When the token reaches 10, you can proceed to the final stage, SETTING IN ORBIT, as your character will have successfully implemented the change towards more physical activity and less sedentary behavior."**

At this stage, the physiotherapist and the participant swap places to facilitate the manipulation of the cards, which also breaks up sedentary behavior.

From there, the participant plays alone and progresses by rolling the dice. The player plays the card corresponding to the emblem on the space where they land. Once they've answered the question on the card, they roll the dice again, and so on.

Based on the participant's responses, the physiotherapist moves the yellow token on the visual scale from 1 to 10. The more the participant finds answers leading to programming physical activity or reducing sedentarity for their character, the more the physiotherapist can progressively move the token towards 10.

This flexibility allows the physiotherapist to manage the pace of the game, moving faster or slower toward 10. The physiotherapist will ensure that the participant has drawn at least one card from each category (Agenda / Resources / Social Support / Weather) before allowing passage to the next stage. If randomness doesn't work well, the physiotherapist can intervene (e.g., switching the card to be drawn).

**GAME PHASE 4: ‘SETTING THE STAGE’ - Anticipating the obstacles to staying on course**

**‘Your character has reached the final stage of the game! In this phase of the game, he will have to avoid the last little traps that life can throw at him, which could reduce his ability to maintain his physical activity over the long term’**

The subject continues to play alone with the die. They now draw from the SUN cards. When the subject lands on the logos, the physio says the following:

**Demotivation: ‘OK, your character has lost his motivation, play a ............ card until his motivation returns!’**

**Illness / Accident: ‘Ouch, your character has been injured and can no longer maintain his AP activity for a while. Play a card ............... and then go back up.’**

When the subject comes across these 2 logos, the player goes back down in the APPROACH phase because the injury, illness or loss of motivation no longer allows him to maintain his AP. He therefore plays 1 more square on the APPROACH circle before going back up to THE SETTING IN ORBIT and continuing to play. The subject must understand that failure is part of the process of changing behaviour and that it must not be dramatised.

All the ‘SUN’ cards must be played, right up to the last one, which invites the subject to set themselves their own challenges in order to move more.

**CONCLUSION - Choosing challenges to move more [15 minutes]**

**Well done, you've succeeded in getting your character to the SUN, i.e. to the long-term maintenance of his new health behaviour. He's moving more and it's going to last!**

**Now it's your turn to challenge yourself to move more. You've already thought about the challenges you could take on between now and our next meeting. You can write these challenges on your dream card. You can set yourself challenges to be more physically active or less sedentary. These challenges must be achievable within the next few days. When you've finished, I'll take a photo and we'll talk again in 2 months' time at your follow-up appointment. And don't hesitate to display your dream card on your fridge!**

The subject unfolds the dream card and completes 1, 2 or 3 challenges that they have set themselves until their next visit. The challenge is to be more physically active and/or less sedentary.

Once the challenges have been chosen and recorded by the subject on the dream card, he or she will note his or her level of confidence by objective on the back of the card. How capable do I feel of meeting this challenge by the next appointment, in two months' time?

At this point, they are considered to have reached the ‘ACTIVE OBJECTIVE’ and can discover what is hidden in the centre of the game: a SUN key ring that they can immediately add to their keys, symbolising the process they have embarked upon.


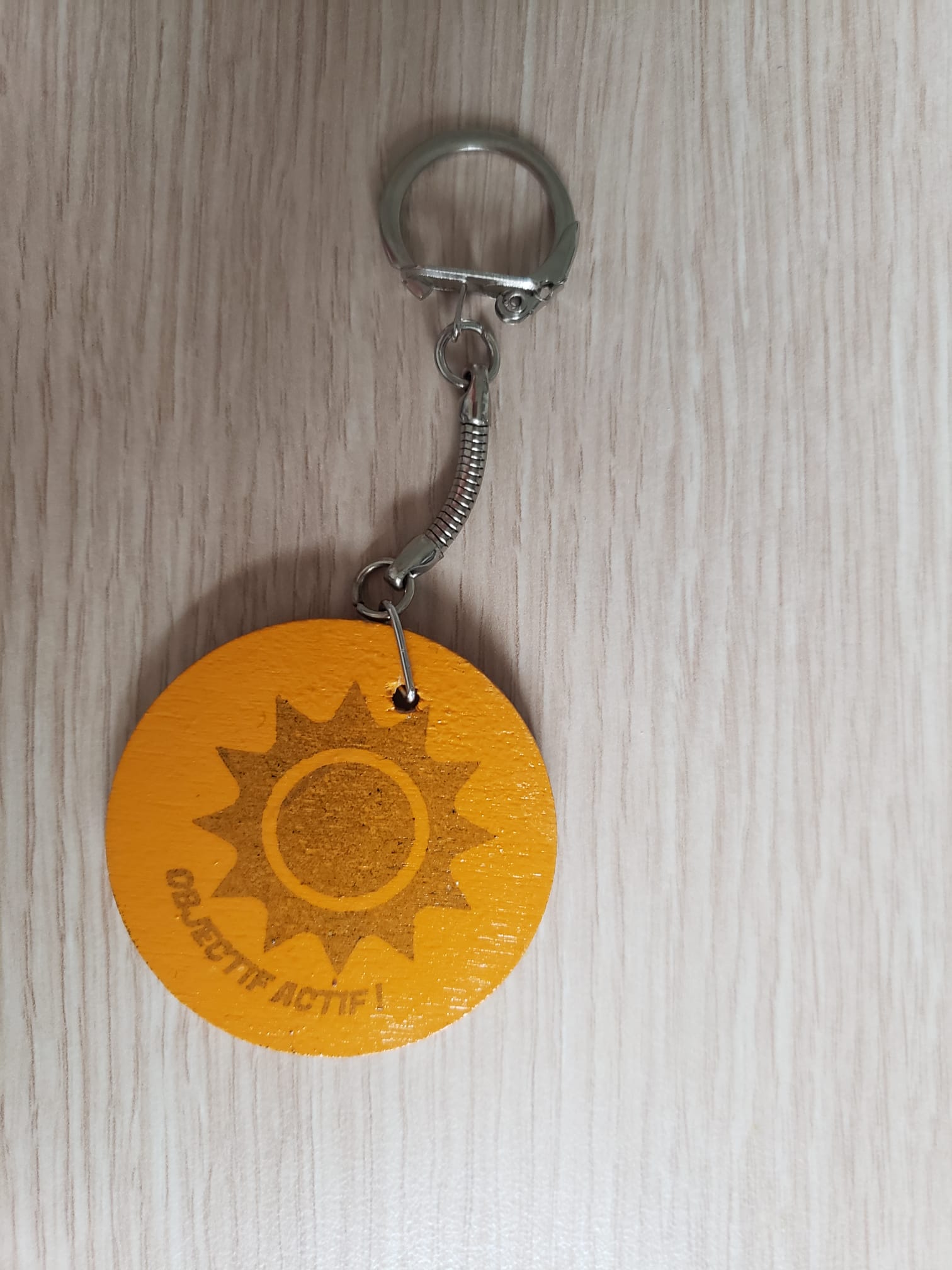


The physio takes photos of the challenges. The participant leaves with their dream card and key ring, which they keep as a reminder of their commitment.
